# Supplementary material for: Molecular architecture and functional dynamics of the pre-incision complex in nucleotide excision repair
Source: Nat Commun. 2024 Oct 1;15:8511. doi: 10.1038/s41467-024-52860-y (PMC11445577; doi:10.1038/s41467-024-52860-y)
Supplement: Supplementary file 1 — Supplementary Information [file 41467_2024_52860_MOESM1_ESM.pdf]

SUPPLEMENTARY INFORMATION

SUPPLEMENTARY NOTES

Supplementary Note 1

TFIIH serves as a molecular ruler defining the spatial extent of the NER bubble

TFIIH interactions are key for determining the optimal size of the NER bubble and the relative positioning of the XPG and XPF nucleases. Both factors are crucial for NER's precise dual incision. Specifically, the lesion containing DNA strand is directed from the 5' junction toward XPD and accommodated within the DNA binding groove of the translocase. The single-stranded DNA exits XPD through a narrow constriction between the iron-sulfur (Fe-S) and Arch domains. The exact footprint of XPD on ssDNA (~12 nucleotides) and the narrow size distribution of post-incision gap products impose stringent limits on the possible length of the NER bubble region. Excision of the damaged DNA segment produces oligonucleotides with an optimal length of 27 nucleotides. Considering the biochemically established incision patterns of the XPG and XPF nucleases – one nucleotide versus 3–5 nucleotides into duplex DNA – the optimal bubble size corresponds to 23 nucleotides. We further confirm that ssDNA length below 21 nucleotides is incompatible with the TFIIH/XPA/DNA cryo-EM structure and our prior model of the lesion scanning complex. Moreover, biochemical constraints impose an upper limit on the NER bubble size of ~30 nucleotides. Thus, our hybrid model of the pre-incision complex features 23 nucleotides between the 3' and 5' junctions, matching the most probable length of the excision products (**Fig. 1a**). This optimal length precisely aligns with the path of the damaged strand through XPD and XPG, while the undamaged strand conforms to the expected footprint of RPA on ssDNA.

Supplementary Note 2

TFIIH serves as a rigid platform for the recruitment of XPG and XPF/ERCC1 within the PInC

To compare the functional dynamics of NER complexes at different stages of the pathway, we perform microsecond-timescale molecular dynamics simulations of PInC, LSC and apo-TFIIH. We compare the relative flexibility of these complexes by mapping computed B-factors from the simulations onto the corresponding structural models (**Supplementary Fig. 6**).

TFIIH in the pre-unwound complex closely resembles apo-TFIIH. Thus, we use the MD conformational ensemble of apo-TFIIH as a proxy to draw conclusions about the functional dynamics of the pre-unwound complex. In this context, the XPB and XPD TFIIH subunits are spaced apart by the presence of MAT1. The more open, crescent-like TFIIH subunit arrangement results in greater

overall flexibility, as evidenced by the higher B-factor values (**Supplementary Fig. 6a** and **6d**). Notably, the XPB ATPase modules remain dynamically independent due to the relatively mobile XPB-XPD interface. Since XPD binds only one of XPB's RecA domains, the second domain is free to swing and push on duplex DNA (**Supplementary Fig. 6d**). Thus, XPB's dsDNA translocase activity is intact. Notably, DNA unwinding by XPB is needed in early NER to expand the nascent bubble. XPD's Arch and Fe-S domains also remain mobile (**Supplementary Fig. 6d**), suggesting XPD retains its internal dynamics.

By contrast, in the lesion scanning complex XPA replaces MAT1, forcing XPB, XPD and p44 to come together and strengthen their interfaces. As the XPB–XPD gap closes, TFIIH adopts a locked circular shape, which imparts rigidity to the LSC. This results in lower computed B-factors over the assembly (**Supplementary Fig. 6b** and **6e**). In this arrangement, XPD engages both ATPase modules of XPB (**Supplementary Fig. 6e**), blocks dsDNA unwinding, and stops further expansion of the NER bubble. Importantly, the XPD Arch and Fe-S domains are mobile (**Supplementary Fig. 6e**), suggesting intact ssDNA translocase activity. The opening/closing domain dynamics is key for XPD's ability to move ssDNA through its narrow DNA-binding groove to scan for bulky lesions.

TFIIH mobility is even further reduced in the PInC, yielding exceptionally low B-factors (**Supplementary Fig. 6c** and **6f**). We observe a ridge of stability that extends from the XPG core, encompasses TFIIH's XPD, XPB and p44 subunits, most of RPA, XPA's DBD and extended helix domains, and XPF/ERCC1. Limited mobility is seen in the TFIIH lever arm (comprised of p52, p8, p34, p62), part of XPG's catalytic core binding the 3' duplex, the mobile RPA32D domain, XPA's N-terminal helix and C-terminal β-sheet. Remarkably, the internal dynamics of XPD, including the opening and closing motions of the Arch and Fe-S domains, are completely suppressed (**Supplementary Fig. 6f**), effectively precluding scanning for DNA lesions. Structural rigidity is enhanced by the insertion of the XPG anchor and coiled-coil helices into the XPD DNA-binding groove, capping ssDNA and serving as a molecular wrench to block DNA translocation. Thus, we conclude that the NER machinery undergoes progressive loss of mobility transitioning from DNA-unwinding to lesion scanning and strand incision. Specifically, the loss of XPD's residual mobility converts PInC into a rigid platform for the assembly of XPF/ERCC1 and XPG.

SUPPLEMENTARY FIGURES

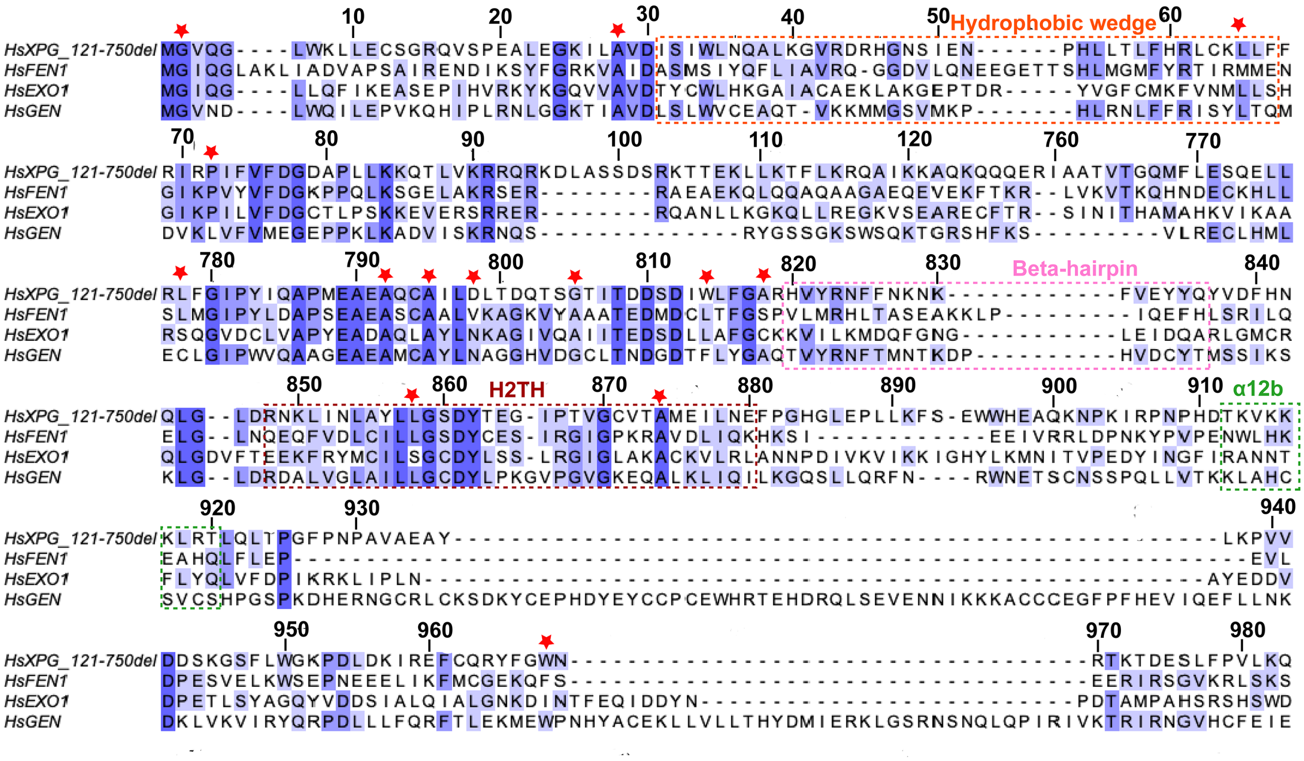

Supplementary Figure 1. Sequence alignment of XPG catalytic core with FEN1, EXO1, and GEN1. Conserved structural motifs are highlighted by dashed-line boxes. Additional conserved regions are shaded light blue (moderately conserved) or dark blue (strongly conserved). Positions of disease mutations are indicated by red stars.

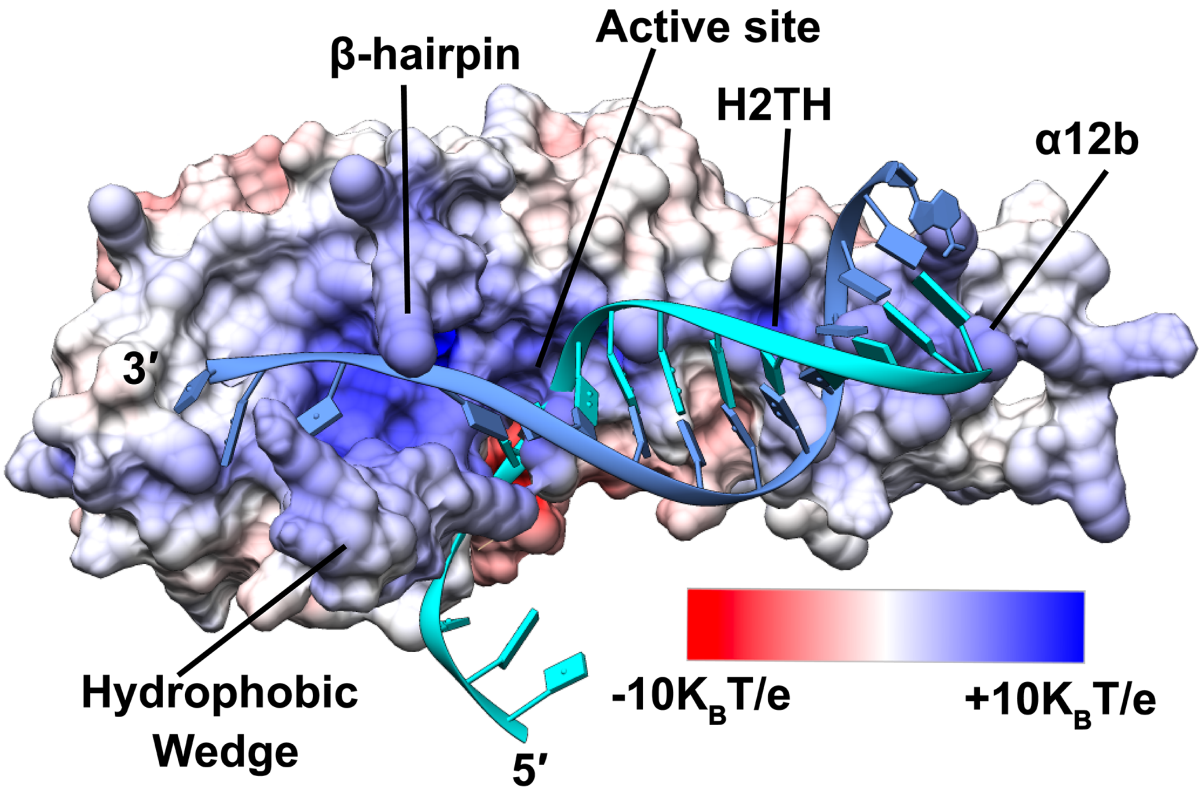

Supplementary Figure 2. XPG exhibits a conserved electrostatically compatible surface for recognizing the dsDNA. The electrostatic potential was mapped onto the molecular surface and colored from red (negative) to blue (positive). Conserved structural motifs in the XPG catalytic core are labelled and their positions indicated by black lines.

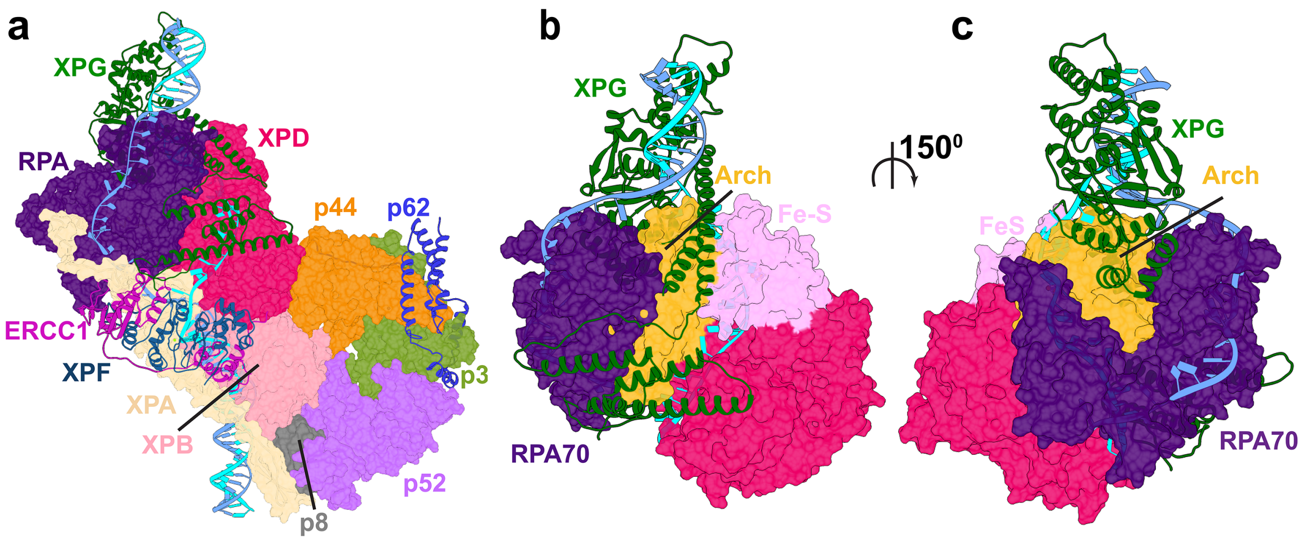

Supplementary Figure 3. Alternative model of PInC with tilted XPG core in contact with the XPD Arch domain. a, View of PInC complex colored by subunits. XPG, XPF/ERCC1, partial p62 (three helix bundle) and DNA are depicted in cartoon representation. The TFIIH subunits (except p62), XPA, RPA, and DNA are shown in surface representation. The damage DNA strand is colored in cyan, and the non-damaged strand is in blue. **b,** Close-up view of XPG positioned at the 3' DNA junction, establishing contacts with the XPD Arch domain. XPG is depicted in both cartoon representations, colored in dark green. **c,** Rotated close-up view of XPG interacting with the Arch domain.

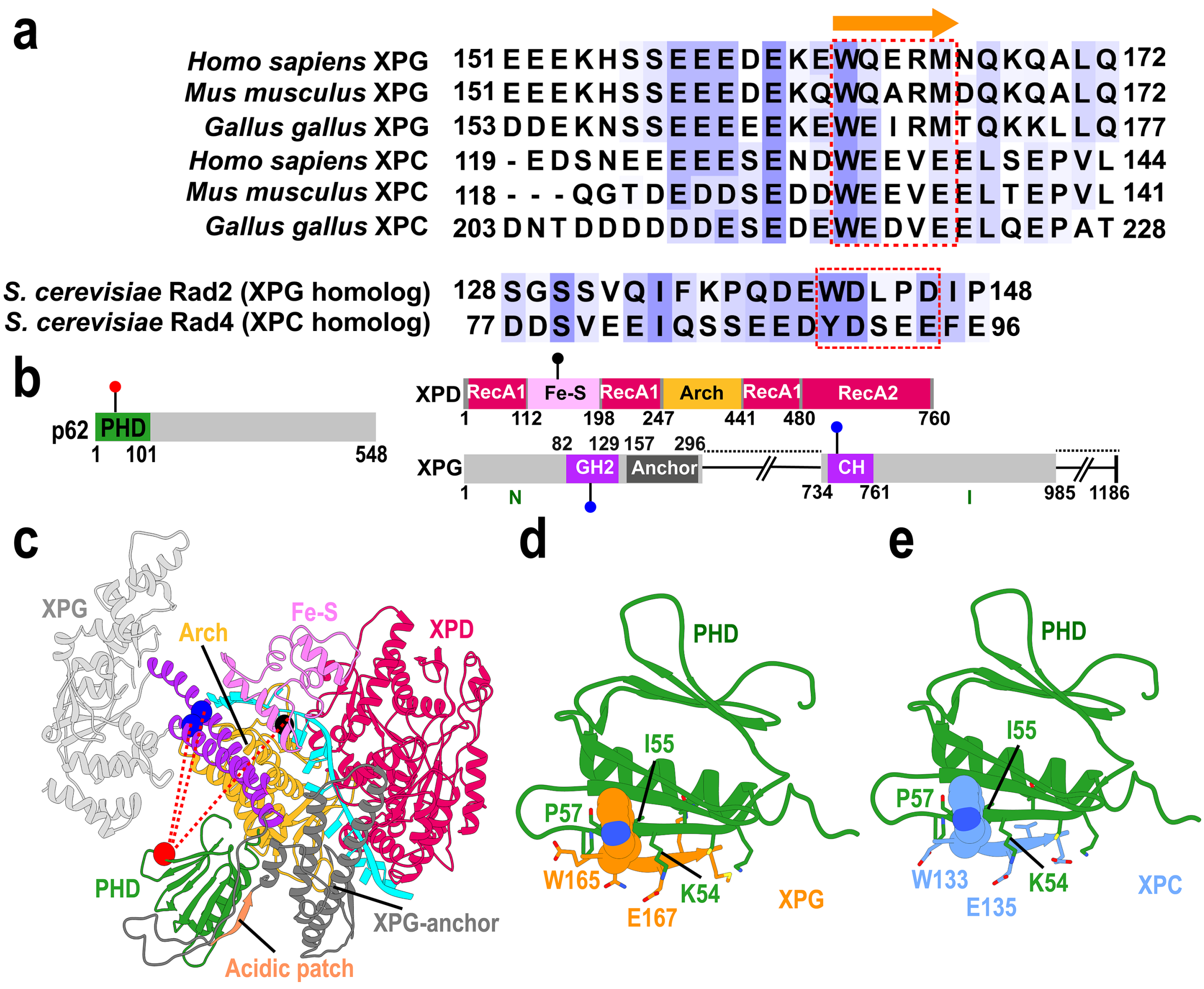

Supplementary Figure 4. XPG and XPC compete for binding to the p62 PH domain of TFIIH. a, Sequence alignment of acidic patches in XPC and XPG predicted to bind p62's PH domain. Sequences from three vertebrates and yeast are included in the alignment**.** Conserved regions are highlighted in light blue (moderately conserved) or dark blue (strongly conserved). **b,** Crosslinks between PHD and the XPD Arch and Fe-S domains mapped onto the p62, XPD and XPG sequences, respectively. **c,** View of the XPG anchor domain (gray) interacting with p62's PH domain (green) through its acidic patch (orange). Crosslinks from the p62's PH domain to the XPD Arch (blue spheres) and Fe-S domains (black sphere) are shown by red dashed lines. **d,** p62's PH domain (green) interacting with the XPG acidic patch (orange). Interacting residues along the antiparallel β-sheet are shown in stick representation. The W165 residue is shown sphere representation. **e,** p62's PH domain (green) interacting with the XPC acidic patch (blue). Interacting residues along the antiparallel β-sheet are shown in stick representation. The W133 residue is shown sphere representation.

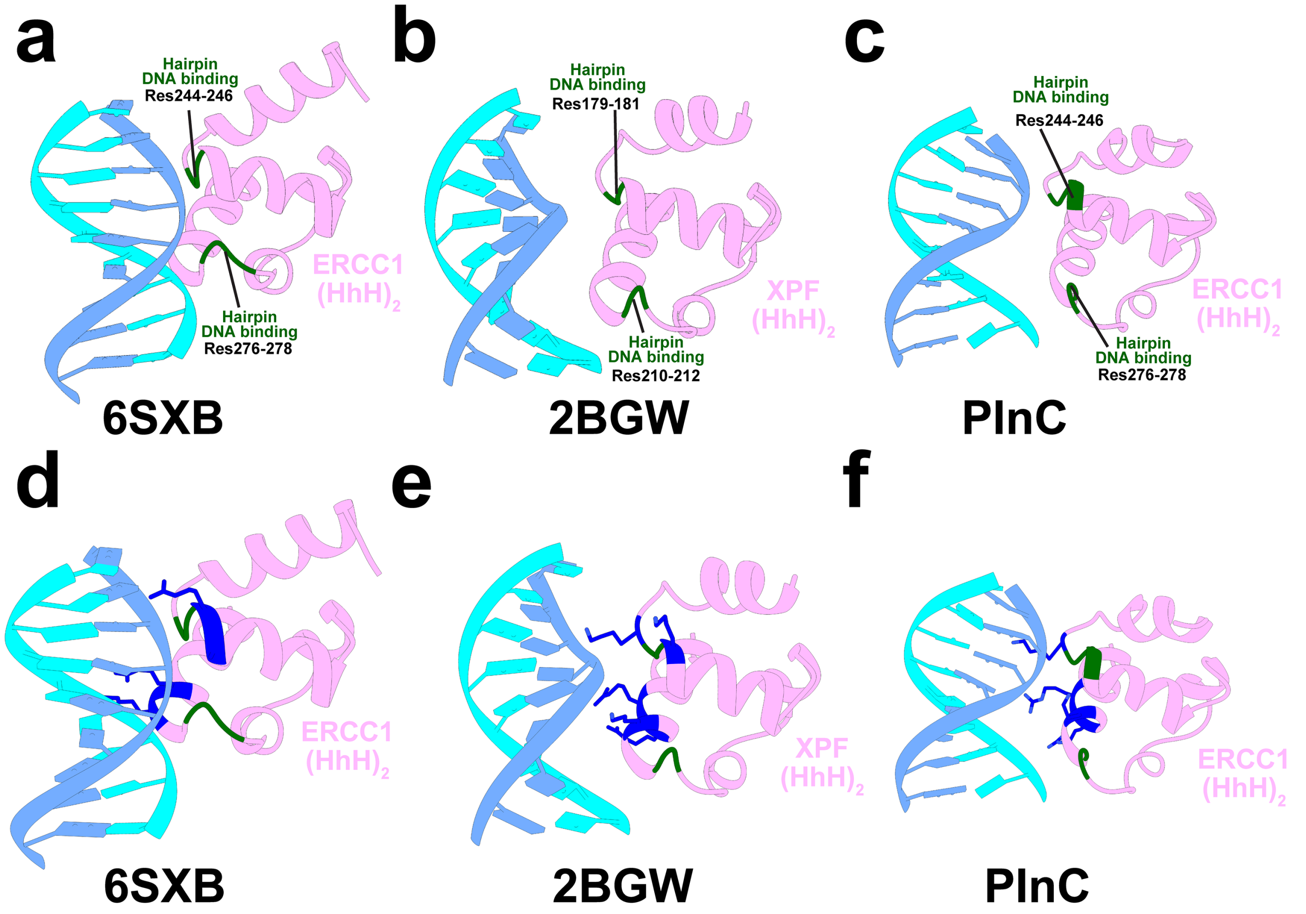

Supplementary Figure 5. ERCC1 (HhH)$_2$ binding mode to duplex DNA Two hairpin loops are involved in DNA binding in **a.**, XPF/ERCC1/DNA cryo-EM structure **b.**, *Aeropyrum pernix* SNF2 structure **c.**, our PInC model. Detailed interactions at the interface of ERCC1 (HhH)$_2$ with dsDNA in **a.**, XPF/ERCC1/DNA cryo-EM structure **b.**, *Aeropyrum pernix* SNF2 structure **c.**, our PInC model. Arg and Lys residues contacting the DNA backbone are shown in stick representation and colored in blue.

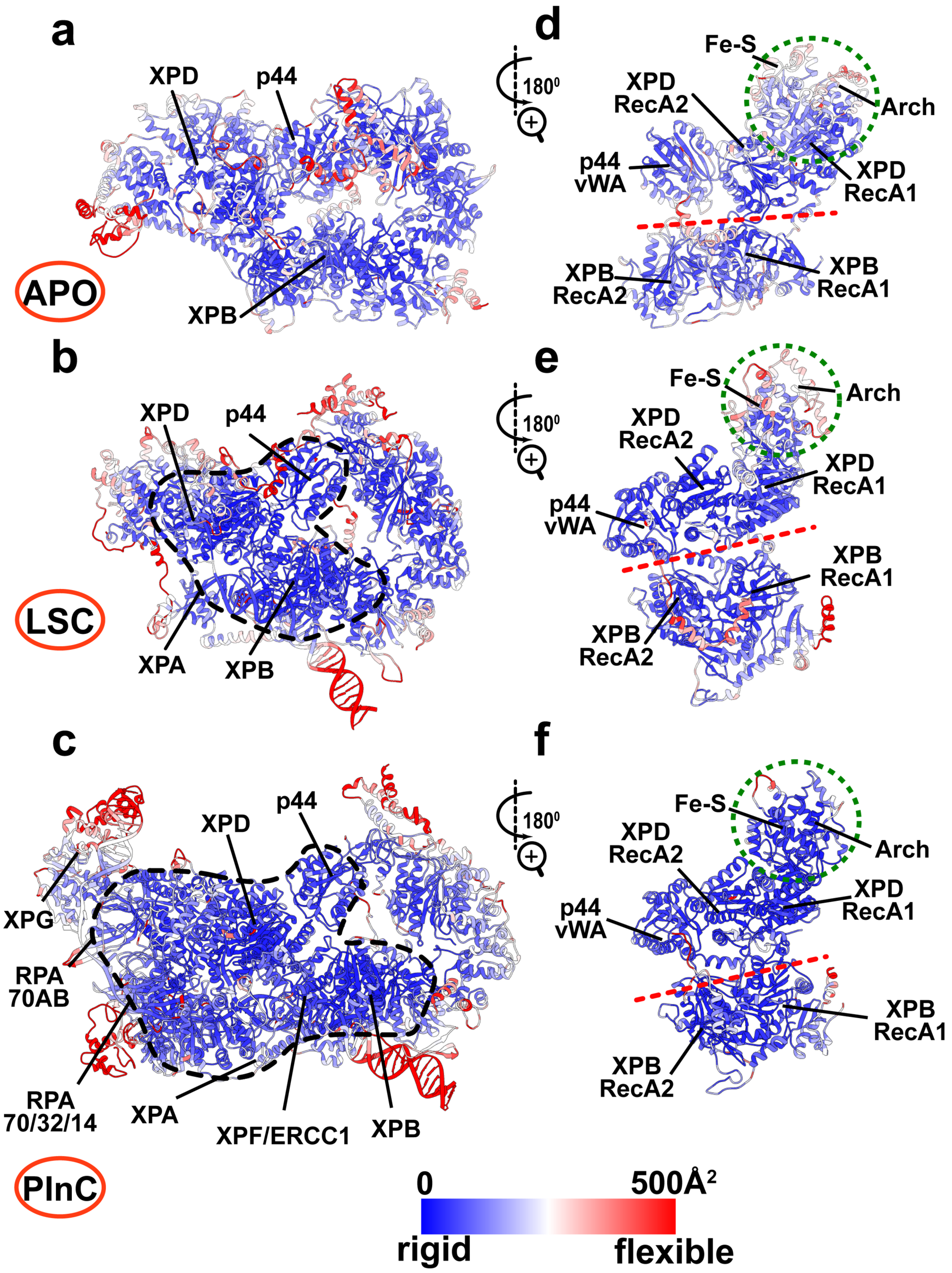

Supplementary Figure 6. NER machinery experiences progressive loss of mobility transitioning from DNA-unwinding to lesion scanning and strand incision. Computed B-factors mapped onto the structural models of **a**, apo-TFIIH, **b**, LSC **c**, PInC. B-factor values are colored from low (blue) to high (red). Close-up views of the rigid (blue) versus flexible (red) elements at the XPB-XPD interface in **d**, apo-TFIIH, **e**, LSC and **f**, PInC. Black dashed outline highlights a ridge of structural stability that expands in the transition from the LSC the to the PInC. The XPB–XPD interface is highlighted by a red dashed. The interface of XPD's Fe-S and Arch domains is outlined by a green dashed circle.

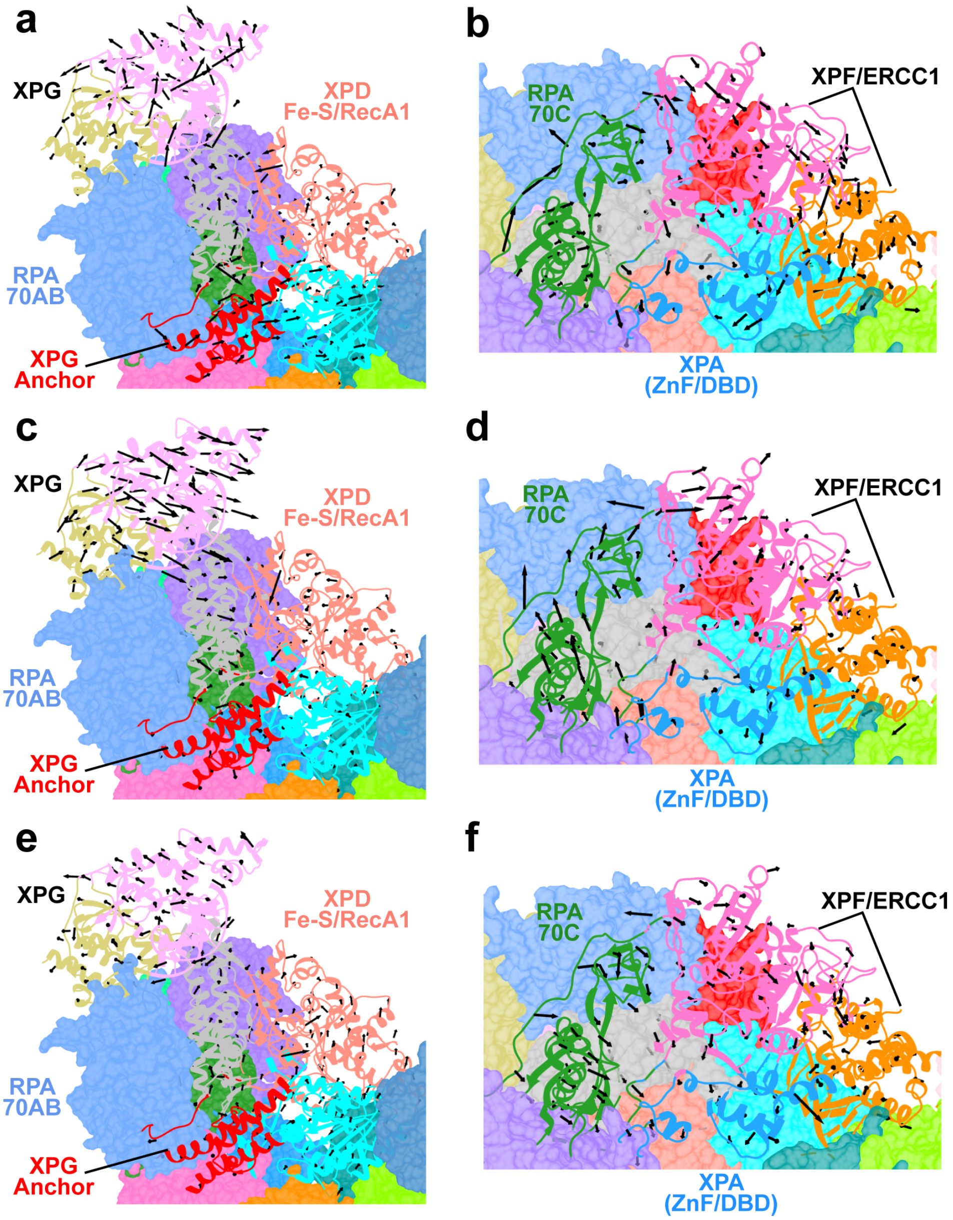

Supplementary Figure 7. Mapping of global motions onto PInC's community structure shows DNA translocation is suppressed for both XPD and XPB. First principal mode of PInC is shown in **a**, close-up view of the motions of dynamic communities near XPD; and **b**, close-up view of the motions of communities near the 5' junction. Second principal mode of PInC is shown in **c**, close-up view of the motions of dynamic communities near XPD; and **d**, close-up view of the motions of communities near the 5' junction. Third principal mode of PInC is shown in **e**, close-up view of the motions of dynamic communities near XPD; and **f**, close-up view of the motions of communities near the 5' junction. Gray arrows indicate the directionality of the motions of the Cα atoms of the assembly. Communities are colored the same as in Fig. 6.

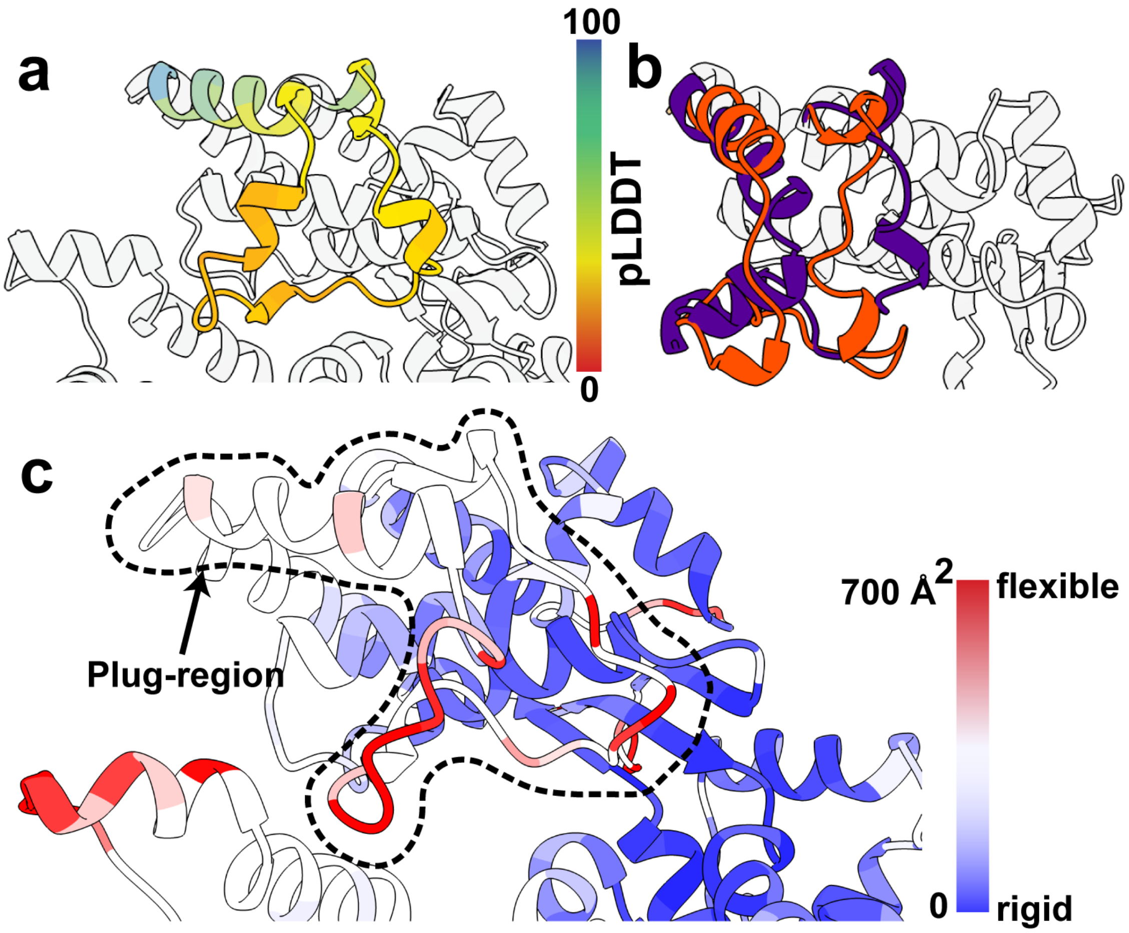

Supplementary Figure 8. a) XPD-plug region modeled by AlphaFold2 with pLDDT score color-coded onto the structure; b) Two conformations of the XPD-plug region from MD simulations of XPD, showing mobility and partial unfolding; c) B-factors mapped onto the XPD structure highlight increased flexibility in the XPD-plug region calculated from a 1 μs molecular dynamics simulation of apo-XPD.

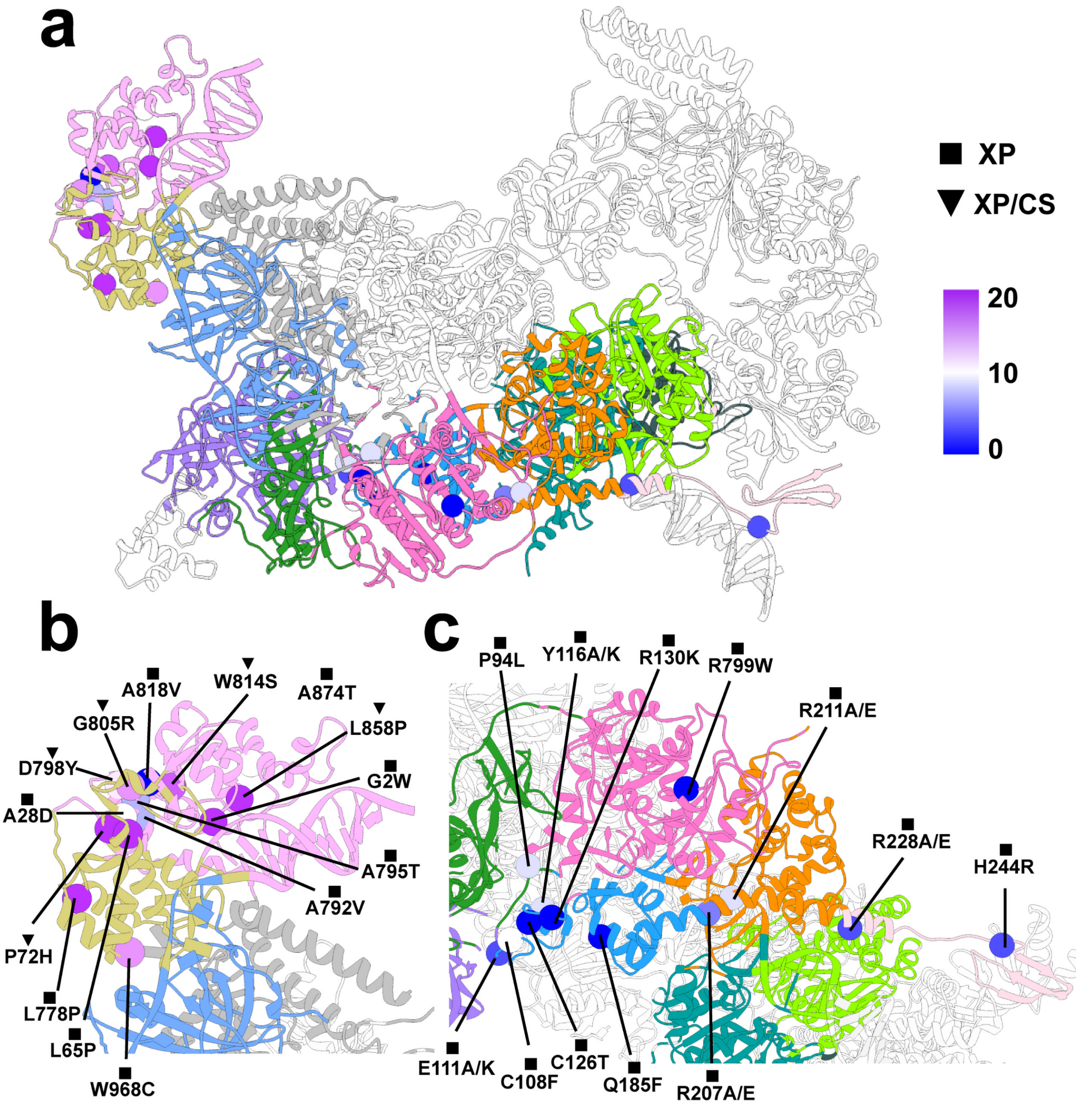

Supplementary Figure 9. Effect of disease mutations on protein stability. **a**, Point mutations in the XPG, XPA and XPF protein chains are mapped onto the PInC model and colored by Rosetta ddG scores. PInC communities are colored according to Fig. 6. Disease phenotypes are labelled as XP or XP/CS and identified by symbols. **b**, Close-up view of mutations within XPG; **c**, Close-up view of mutations in XPA.

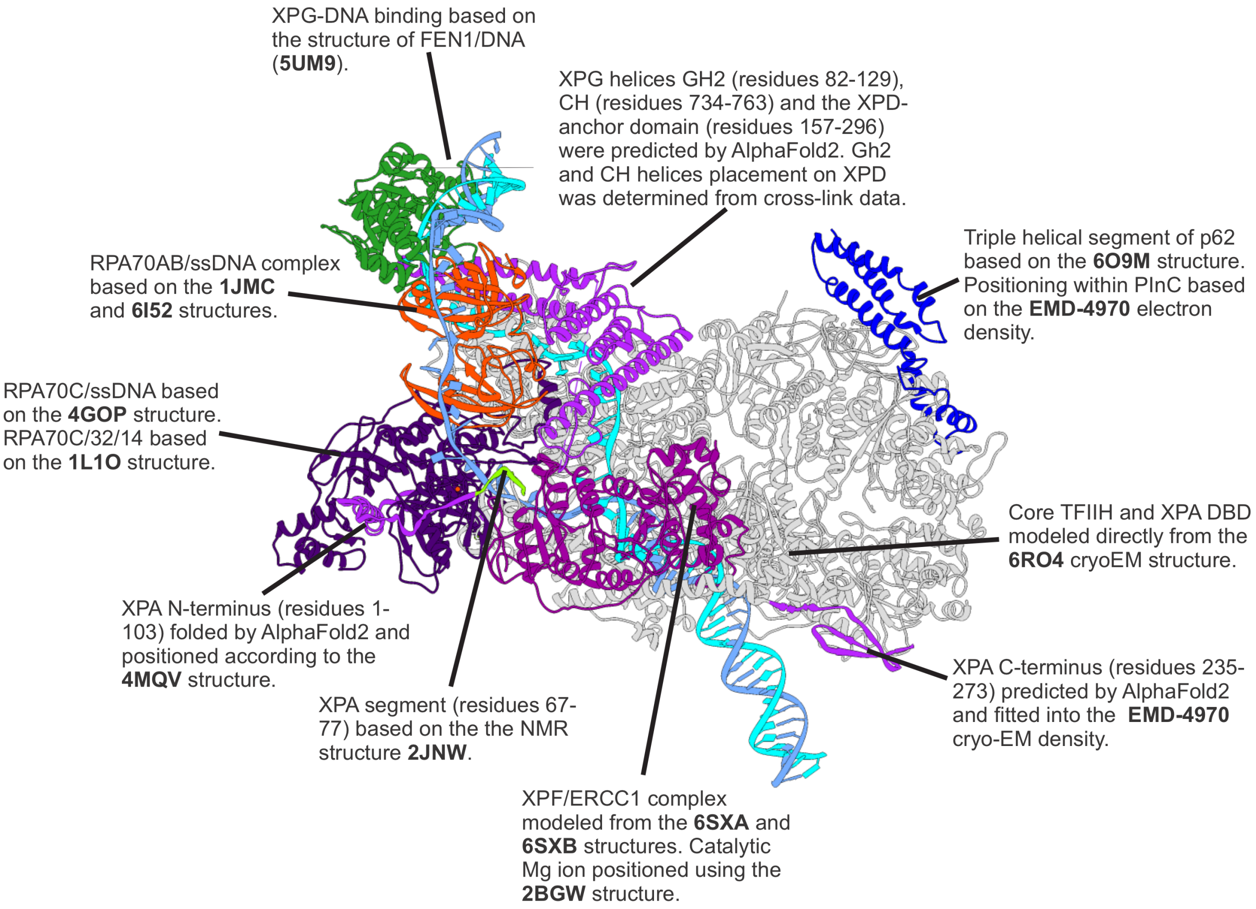

Supplementary Figure 10. Sources of experimental structural information used in constructing the integrative model.

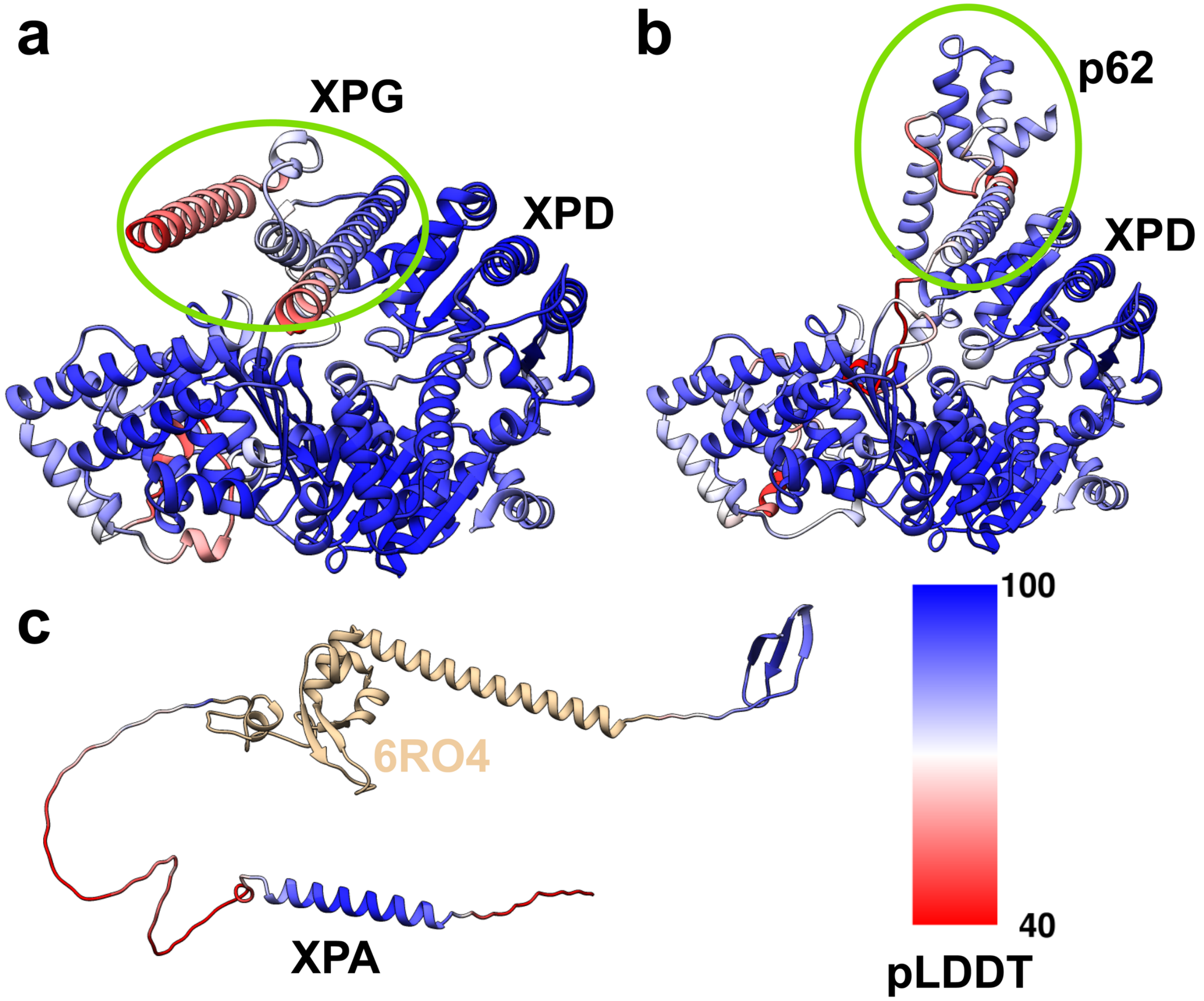

Supplementary Figure 11. Regions of the PInC assembly modeled with AlphaFold2 with pLDDT scores mapped onto the structures. a) XPG-anchor–XPD complex; b) p62 BSD2 and XPD-anchor in complex with XPD; c) XPA C-terminal and N-terminal regions.

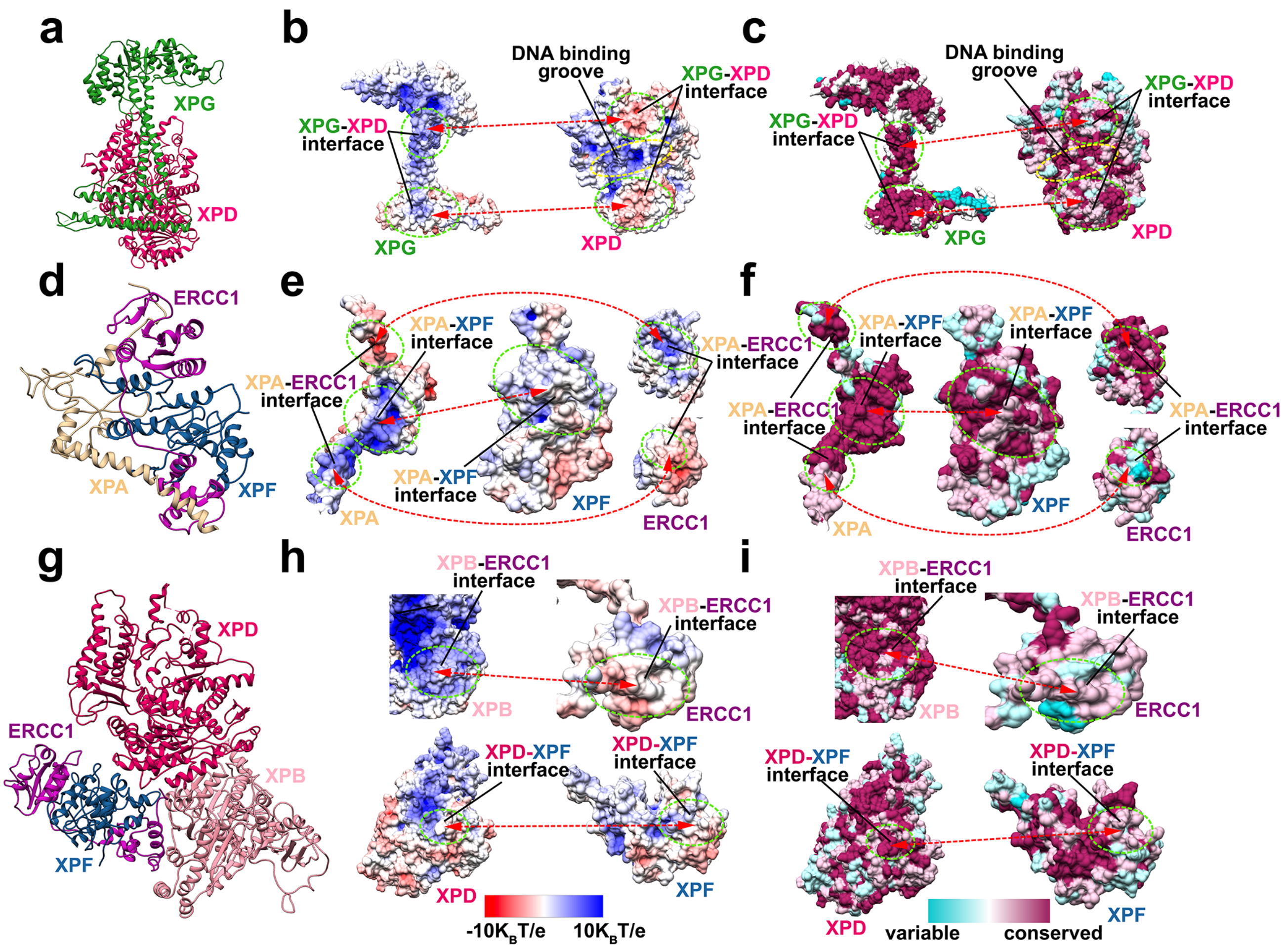

Supplementary Figure 12. Newly modeled protein interfaces of the PInC integrative model showing geometric and electrostatic complementarity and sequence conservation.

SUPPLEMENTARY TABLES

Protein	Mutation	Disease*	Community Resides In	Community Interface(s)	ΔΔG Score
XPA	P94L	XP	T	T/O	8.726
XPA	C108F	XP	T	Non-interfacial	9.751
XPA	C108Y	XP	T	Non-interfacial	19.671
XPA	E111A	XP	C	C/T	1.749
XPA	E111K	XP	C	C/T	1.002
XPA	Y116A	XP	T	T/O	6.923
XPA	Y116K	XP	T	T/O	7.824
XPA	C126T	XP	T	Non-interfacial	-2.306
XPA	R130K	XP	T	Non-interfacial	-0.483
XPA	Q185F	XP	T	Non-interfacial	-0.698
XPA	Q185H	XP	T	Non-interfacial	0.981
XPA	R207A	XP	N	N/F, N/T	-0.626
XPA	R207E	XP	N	N/F, N/T	4.414
XPA	R211A	XP	N	N/F	2.092
XPA	R211E	XP	N	N/F	6.246
XPA	R228A	XP	Q	N/Q, N/K	0.14
XPA	R228E	XP	Q	N/Q, N/K	1.196
XPA	H244R	XP	Q	Non-interfacial	0.347
XPF	R799W	XP	F	Non-interfacial	-4.103
XPG	G2W	XP	M	Non-interfacial	33.206
XPG	A28D	XP	R	R/M	9.795
XPG	L65P	XP	R	Non-interfacial	35.447
XPG	P72H	XP/CS	R	Non-interfacial	20.87
XPG	L778P	XP	R	Non-interfacial	34.216
XPG	A792V	XP	M	Non-interfacial	6.471
XPG	A795T	XP	M	M/R	5.174
XPG	D798Y	XP/CS	M	M/R	12.316
XPG	G805R	XP/CS	R	R/M	14.107
XPG	W814S	XP/CS	M	Non-interfacial	17.917
XPG	A818V	XP	M	Non-interfacial	-0.556
XPG	L858P	XP/CS	M	Non-interfacial	28.82
XPG	A874T	XP	M	Non-interfacial	10.779
XPG	W968C	XP	R	R/I	14.176

*Disease abbreviations: Xeroderma pigmentosum (XP) and Xeroderma pigmentosum/Cockayne syndrome (XP/CS)

Supplementary Table 1. **Patient Derived Mutations within XPA, XPF, and XPG**. Dynamic community identity, community interface information and Rosetta DDG scores are provided for each mutation.

Protein	Chain	Size (aa)	Modeled Residues	Alternative names	Structures (PDB IDs) used for hybrid modeling[a]
XPB	A	782	34-203,248-720	ERCC3	Modeled from 6RO4
XPD	B	760	1-760	ERCC2	Modeled from 6RO4
p52	C	462	18-458	GTF2H4	Modeled from 6RO4
p44	D	395	11-387	GTF2H2	Modeled from 6RO4
p34	E	308	1-292	GTF2H3	Modeled from 6RO4
p8	F	71	2-67	GTF2H5	Modeled from 6RO4
XPA	G	273	1-273		Residues 104-234 modeled from 6RO4; The XPA N-terminal extension (residues 1-103) and the C-terminal extension (β-domain) (residues 235-273) modeled using AlphaFold2; Residues 22-40 was positioned by the X-ray structure of RPA32C/Smarcal1 N-terminus (4MQV). Residues 67-77 modeled from NMR structure (2JNW)
p62	H	548	395-548	GTF2H1	Modeled from 6O9M and cryo-EM density (EMD-4970)
XPG	I	1186	1-296,733-985	ERCC5	DNA-bound XPG modeled based on the human FEN1/DNA X-ray structure (5UM9). The two XPG gateway helices (GH2 residues 82-129) and the capping helix (CH, residues 734-763) were predicted with AlphaFold2 and positioned in the gap between XPD's Arch and Fe-S domains in accordance with the crosslink data. The XPD-anchor domain (residues 157-296) was predicted by AlphaFold2 and positioned based on TFIIH/XPA/DNA cryo-EM density (EMD-4970).
XPF	J	916	680-906	ERCC4	Modeled from cryo-EM structures of XPF/ERCC1 (6SXA and 6SXB). Mg^{2+} ion coordination was based on the *Aeropyrum pernix* SNF2 structure (2BGW).
ERCC1	K	297	100-297		Modeled from cryo-EM structures of XPF/ERCC1 (6SXA and 6SXB).
RPA70	L	616	183-616		RPA70AB/ssDNA (residues 183-415) was modeled by the yeast RPA/ssDNA structure (1JMC) and human apo-RPA 70AB (6I52). RPA70C/ssDNA (residues 442-596) was modeled by *Ustilago maydis* RPA/ssDNA structure (4GOP) and human trimer core structure (1L1O).
RPA14	M	121	3-117		Modeled from human trimer core structure (1L1O).
RPA32	N	270	44-268		Modeled from human trimer core structure (1L1O).

a. Any missing residues in the structure were built with the Modeller software package. Corrections in TFIIH were made based on 6O9M.

Supplementary Table 2 Summary of PInC structural elements and original sources used for hybrid modeling
