# Supplementary material for: Molecular architecture and functional dynamics of the pre-incision complex in nucleotide excision repair
Source: Nat Commun. 2024 Oct 1;15:8511. doi: 10.1038/s41467-024-52860-y (PMC11445577; doi:10.1038/s41467-024-52860-y)
Supplement: Supplementary file 3 — Description of Additional Supplementary Files [file 41467_2024_52860_MOESM3_ESM.pdf]

Description of Additional Supplementary Files

File Name: Supplementary Data 1
Description: **The final configuration of the PInC molecular dynamics trajectory provided in PDB format.**

File Name: Supplementary Movie 1
Description: **Structural organization of the PInC assembly.** The PInC assembly is colored by subunits. XPG, XPF/ERCC1 and DNA are shown in cartoon representation. TFIIH, XPA and RPA are shown in surface representation. The lesion containing DNA strand is shown in cyan; the undamaged strand is shown in blue.

File Name: Supplementary Movie 2
Description: **Mapping of XP and XP/CS disease mutations onto the PInC community structure.** Dynamic communities from network analysis are colored as denoted in Fig. 6. Human disease mutations are represented as spheres and colored by phenotype.

File Name: Supplementary Movie 3
Description: **Mapping of XP and XP/CS disease mutations onto the PInC community structure.** Dynamic communities from network analysis are colored as denoted in Fig. 6. Human disease mutations are represented as spheres and colored by by Rosetta ddG score – proxy for protein stability.
